# Immediate Effects of Adipose‐Derived Mesenchymal Stem Cell Administration on Cholinesterase Levels and Grip Strength

**DOI:** 10.1002/alz70859_102343

**Published:** 2025-12-25

**Authors:** Kazuo Shigematsu, Naoyuki Komori, Hisakazu Yamagishi

**Affiliations:** ^1^ Nagitsuji Hospital, Kyoto, Kyoto Japan; ^2^ Kyoto Prefectural University of Medicine, Kyoto, Kyoto Japan

## Abstract

**Background:**

Immediate improvements in subjective and objective symptoms can sometimes be observed following the administration of adipose‐derived mesenchymal stem cells (ADSCs). This rapid improvement was surprising, as we had assumed that regenerative medicine would require several days or more to show effects. Therefore, we measured grip strength and cholinesterase values before and after treatment. ADSCs have gained attention in regenerative medicine due to their multipotent nature and potential therapeutic effects.

**Method:**

We conducted a prospective observational study on 21 patients who received ADSC treatment. Informed consent was obtained from all participants. Serum cholinesterase levels and grip strength were measured immediately before and after ADSC administration. Grip strength was measured on the non‐infusion side, maintaining the same posture for both pre‐ and post‐treatment measurements.

**Result:**

The study included patients with various conditions: Parkinson's disease (PD, n=9), Amyotrophic Lateral Sclerosis (ALS, n=4), Alzheimer's disease (n=2), Chronic Obstructive Pulmonary Disease (COPD, n=4), and Progressive Supranuclear Palsy (PSP, n=1). The mean age was 67.5 years (range: 53‐81), with 14 males and 7 females. A paired samples t‐test revealed a statistically significant decrease in cholinesterase levels after ADSC administration (mean difference: ‐21.905 U/L, p < 0.001). Although not statistically significant, there was a trend towards improvement in grip strength (mean difference: 1.0619 kg, p = 0.119).

**Conclusion:**

Our findings demonstrate a significant immediate decrease in serum cholinesterase levels following ADSC administration. The observed decrease in cholinesterase levels might indicate an enhancement of acetylcholine activity, potentially explaining the trend towards improved grip strength. The immediate effects observed in this study are intriguing, as they suggest that ADSCs may have rapid neuromodulatory effects beyond their known regenerative properties. Previous studies have shown that ADSCs can differentiate into various cell types, including neural cells, and secrete neurotrophic factors. The trend towards improved grip strength, although not statistically significant, is noteworthy. Grip strength is a reliable indicator of overall muscle strength and has been associated with various health outcomes. The potential improvement in grip strength immediately after ADSC administration warrants further investigation, as it could have implications for patients with neuromuscular disorders.

